# The Mental Symptoms of Brain Disease

**Published:** 1911-09

**Authors:** 


**The Mental Symptoms of Brain Disease.** By Bernard Hollander, M.D. Pp. 229. London: Rebman Limited.

Dr. Hollander in his preface modestly claims no more than the production of a book which is a summary of existent knowledge as to the functions of the various brain-centres, and a collection of published observations on lesions in relation to these same areas. He has accumulated a large number of cases designed to illustrate the connection of psychical states and capacities with definite brain-centres by means of positive evidence of lesions of them. There is always risk in placing much dependence upon reported cases where their author's tendency may be to endeavour to establish a theory, and thus a good many examples quoted exhibit notable weakness. There is, on the other hand, a large number of cases which in their similarity, and sometimes by the result of operative interference adopted in accordance with the theories advanced, certainly indicate much in support of the views put forward by Dr. Hollander. We quite agree with him that the least satisfactory results in regard to localisation of psychical areas are now obtainable from experiments on animals, and that the main sources of knowledge are from careful clinical observation and punctilious examination after death. It is deplorable in how many of our asylums for the insane the necessity for this is absolutely lost sight of, the case books, except for the statutory entries, being entirely valueless. Thus pathological findings of great interest are without any clinical record.

We hope Dr. Hollander's work may arouse a proper interest in one of the varieties of investigation without which asylum life is profitless and wearisome. The author shows a thorough acquaintanceship with literature dealing with recent advances in our knowledge of the physiology and pathology of the brain-centres, and he has furnished a lucid epitome of this for his readers. We think his enthusiasm at times occasions him to assume too much, and it behoves those who are interested in this matter to pay attention to negative as well as positive findings. From careful observation of brains, running into thousands, we cannot but feel that in many presenting symptoms as well marked and apparently localised as those quoted by the author there are no such marked lesions to be found ; at the same time, we concede that these coarse lesions are only cited to indicate the areas where finer ones may equally be expected.

This book will serve a good purpose in showing the lines upon which further work may be accumulated, and suggesting when and where to look for focal trouble in the brain. The author does not appear to seek much more than this.

